# Efficacy of an experimental toothpaste containing sodium bicarbonate, sodium hyaluronate and sodium fluoride on gingivitis

**DOI:** 10.1186/s12903-024-03981-9

**Published:** 2024-02-09

**Authors:** Alyson Axe, Nisha Patel, Jimmy Qaqish, Martin R. Ling, Mako Araga, Charlie Parkinson, Chaju Ram Goyal

**Affiliations:** 1 Haleon, St George’s Avenue Weybridge, Surrey, ODE KT13 UK; 2 All Sum Research, Mississauga, ON Canada; 3 Haleon, Warren, NJ USA

**Keywords:** Gingivitis, Toothpaste, Plaque, Sodium bicarbonate, Sodium hyaluronate

## Abstract

**Background:**

Gingivitis is driven by plaque accumulation and, if left untreated, can progress to irreversible periodontitis. For many, the mechanical action of toothbrushing does not achieve adequate plaque control. The aim of this study was to investigate whether twice-daily use of a toothpaste containing 0.2% high molecular weight (HMW) sodium hyaluronate with 67% sodium bicarbonate and 0.221% sodium fluoride (experimental toothpaste) could improve gingival health compared with a regular fluoride toothpaste (negative control). The study also assessed whether the experimental toothpaste could provide additive gingival health benefit over a toothpaste containing only 67% sodium bicarbonate and 0.221% sodium fluoride (positive control).

**Methods:**

This was a single-center, examiner-blinded, randomized, clinical study in healthy adults with mild-to-moderate gingivitis. At baseline, after abstaining from toothbrushing for 12 h, prospective participants underwent oral soft tissue (OST) and oral hard tissue examination followed by assessments for gingival inflammation (Modified Gingival Index [MGI]), gingival bleeding (Bleeding Index [BI]), and supra-gingival plaque (Turesky Plaque Index [TPI]). Eligible participants were stratified by gender and baseline number of bleeding sites (low: <45; high: ≥45 bleeding sites). Following randomization, participants underwent prophylactic dental treatment. Participants received a full OST examination, MGI, BI and TPI assessments after 3 days, 1, 2 and 6 weeks of product use.

**Results:**

In total, 110 participants were screened for study entry and all were randomized to receive one of three toothpastes (experimental: sodium hyaluronate, sodium bicarbonate, sodium fluoride; positive control: sodium bicarbonate, sodium fluoride; negative control: regular fluoride toothpaste). For all measures, significant improvements were observed in participants receiving either sodium bicarbonate-containing toothpaste (experimental or positive control) compared with the regular fluoride toothpaste (negative control) at week 6. No significant difference was observed in any assessment or visit comparing the experimental toothpaste with the positive control.

**Conclusions:**

Both the experimental and the positive control toothpastes demonstrated clinically relevant improvements in gingival health compared with a regular fluoride toothpaste (negative control). However, no additional gingival health improvement was observed for the experimental toothpaste compared with the positive control, therefore, no additional gingival health benefit can be attributed to the inclusion of sodium hyaluronate in this formulation.

**Trial registration:**

ClinicalTrials.gov Identifier: NCT04737538 (04/02/2021).

**Supplementary Information:**

The online version contains supplementary material available at 10.1186/s12903-024-03981-9.

## Background

Oral diseases affect approximately 3.5 billion people worldwide; nearly half of the global population [1]. Gingivitis is a common periodontal disease that develops when dental plaque collects on teeth and along the gingival margin [2–4]. This deposit of bacteria elicits a local inflammatory response in the gingivae [5, 6]. If left untreated, gingivitis can progress to the irreversible phase of periodontitis, where inflammation extends to the underlying tissues, periodontal ligament, and alveolar bone, which can eventually lead to tooth loss [7]. Treatment of gingivitis can, therefore, prevent the progression to periodontitis. Despite being largely preventable with effective plaque control, the incidence of periodontal disease remains high [1, 3–6]. Many people are unable to achieve adequate plaque control by toothbrushing alone, therefore, the effects of toothbrushing can be augmented by using a toothpaste with ingredients to facilitate plaque removal and/or including dental flossing as an adjuvant to toothbrushing [3, 8, 9].

Clinical studies have previously shown that the addition of sodium bicarbonate to a fluoride toothpaste, used twice daily, could reduce gingival bleeding in adults with gingivitis, compared with a toothpaste containing 0% sodium bicarbonate [10–14]. Evidence also suggests that toothpastes including sodium bicarbonate enhance plaque removal [11–13, 15–19]. It is proposed that sodium bicarbonate softens the plaque biofilm, making it easier for the action of toothbrushing (and toothpaste) to physically displace plaque from the tooth surface [15, 20].

Hyaluronic acid (and its sodium salt, sodium hyaluronate) is employed in a number of oral care products [21, 22]. It is a polysaccharide present in the extracellular matrice of many tissues, including periodontal tissues. Hyaluronic acid is highly hygroscopic, allowing it to attract and retain water [23]. In nature, hyaluronic acid is active in maintenance of the elastoviscosity of liquid connective tissue, tissue hydration and water transport, supramolecular assembly of proteoglycans in the extracellular matrix, and facilitation of cell migration [24, 25].

High molecular weight (HMW) sodium hyaluronate has been reported to inhibit bacterial adhesion to dental hard tissue by forming a layer over the surface and thereby reducing the rate of plaque build-up [26, 27]. Studies have reported efficacy of 0.2% weight by weight (w/w) HMW sodium hyaluronate, a primary film-forming polymer in a gel format, in the treatment of gingivitis when used as an adjunct to regular toothbrushing [28–33]. However, 0.2% w/w HMW sodium hyaluronate has not previously been investigated in a toothpaste format. Mechanisms have been postulated, such as the reduction of cell proliferation in gingival epithelial cells, arresting the inflammatory process, however, evidence is limited [34]. As such, further investigation into the potential additional benefit of a toothpaste containing 0.2% w/w HMW sodium hyaluronate for reduction of gingival inflammation and bleeding compared with regular fluoride toothpaste is warranted [34].

As plaque removal is the most important aspect for improving gingival health, this study aimed to explore if there was any additive benefit of combining sodium hyaluronate with the effective plaque removal properties of sodium bicarbonate. Due to the different mechanisms of sodium bicarbonate (that facilitate plaque removal) and hyaluronic acid (that inhibit plaque accumulation), it was hypothesized that, when combined in toothpaste, these mechanisms will be complementary and may result in greater improvements in gingival and plaque control than plaque removal alone. The aim of this proof-of-principle study was to investigate whether an experimental toothpaste, containing 0.2% w/w HMW sodium hyaluronate with 67% w/w sodium bicarbonate and 0.221% w/w sodium fluoride, when used twice daily, could reduce gingival inflammation and bleeding compared with a regular fluoride toothpaste. In addition, this study aimed to assess whether the experimental toothpaste could provide greater benefit over a toothpaste containing only 67% w/w sodium bicarbonate and 0.221% w/w sodium fluoride.

## Methods

This was a single-center, examiner-blind, randomized, stratified, three-treatment, parallel group, clinical study in healthy adult volunteers with mild-to-moderate gingivitis. The study was evaluated and approved by an institutional review board in accordance with the International Council for Harmonization of Technical Requirements for Registration of Pharmaceuticals for Human Use Good Clinical Practice (ICH E6 R2; guidelines for good clinical practice CPMP/ICH/135/95) and applicable country-specific requirements (including the US 21 Code of Federal Regulations 312.3(b) for constitution of independent ethics committees).

### Eligibility criteria

Written informed consent was obtained from each participant prior to the study. The inclusion and exclusion criteria for this study were assessed via gross visual examination by an experienced, suitably trained and calibrated dental examiner or clinically qualified designee.

People with visual signs that could indicate active periodontitis (where inflammation extends beyond the gums to the underlying tissues, periodontal ligament and alveolar bone) in the opinion of the examiner (such as tooth mobility and pocket depth > 6 mm) were excluded from the study to ensure that all participants were periodontally healthy, and no efficacy or safety data would be compromised.

Participants eligible for this study were between the ages of 18 to 65 years, in good health with no significant/relevant medical or oral/dental conditions and mild-to-moderate plaque-induced gingivitis. The defined level of gingivitis at baseline helped to minimize the risk of an atypical or potential lack of treatment response for participants with high levels of gingivitis that could otherwise be managed professionally rather than solely via home use of a twice-daily dentifrice. Gingivitis was assessed based on established clinical measures including the Modified Gingival Index (MGI) and Bleeding Index (BI), which measure gingival inflammation and gingival bleeding, respectively (i.e., gingival health), and the Turesky Plaque Index (TPI) that measures supragingival plaque accumulation [35–37]. At screening (Visit 1), participants were required to have ≥ 20 natural, permanent teeth with ≥ 40 evaluable surfaces for MGI, BI, and TPI Key inclusion criteria were a mean whole mouth MGI between 1.75 and 2.30, a mean whole mouth TPI score ≥ 1.5 and ≥ 20 bleeding sites, which were assessed at baseline examination (Visit 2). Participants who were taking any antibiotic, anti-inflammatory or any systemic medications that could affect gingival condition/bleeding were excluded. This included those who had used an antibacterial toothpaste, mouthwash or any oral care product that could have interfered with plaque formation or measures of gingivitis. Additional medical exclusion criteria included: participation in another clinical trial within 30 days of screening; pregnancy; breast feeding; intolerance/hypersensitivity to study materials; current smoker, or who had stopped within 6 months of screening; current user of any smokeless form of tobacco; any history of alcohol/substance abuse; medical condition which could have compromised study outcomes; or a tongue or lip piercing. Dental exclusion criteria included: active periodontitis and/or treatment for periodontal disease (including surgery) within 12 months of screening; active caries; partial/full dentures; orthodontic appliance; orthodontic therapy within 12 months of screening; and teeth whitening treatment or dental prophylaxis within 12 weeks of screening.

### Study design

Eligible participants were randomly assigned to one of the three experimental groups of 40 participants each using Interactive Response Technology (IRT, a comprehensive randomization and trial supply management system that managed randomization of patients into the various treatment arms using the trial protocol method of randomization to sort patients evenly). The study was examiner-blind, and participants, investigator, clinical examiners, statistician, other employees of the sponsor and vendors acting on behalf of the sponsor were blinded to the product allocation. To ensure the examiner remained blinded, staff involved in the preparation and dispensing of study products worked in a separate area. Study products were dispensed to participants in a blinded fashion, and participants were instructed not to remove study products from the opaque bags while at the study site. Dispensing staff were not involved in any efficacy or safety assessment procedures during the study. Participants were stratified at the point of randomization based on their gender and number of bleeding sites (NBS; low: < 45; high ≥ 45) at baseline. The participants received one of three toothpastes: experimental product (experimental toothpaste containing 67% w/w sodium bicarbonate, 0.2% w/w sodium hyaluronate, and 0.221% w/w sodium fluoride), positive control (toothpaste containing 67% w/w sodium bicarbonate and 0.221% w/w sodium fluoride), or negative control toothpaste containing 1100 ppm fluoride as sodium fluoride (Crest Cavity Protection). All participants were instructed to dose a full ribbon of toothpaste to cover the head of the toothbrush provided (Oral-B Sensi-soft manual toothbrush) and brush for 1 timed minute, twice daily, to reflect the typical brushing time for manual toothbrush users [38]. To standardize compliance, all participants received a brushing instruction/diary sheet and were instructed to record the date and time of each brushing performed during the treatment period, including any missed or additional brushings. Participants also attended each study visit with all tubes of toothpaste provided (used and unused) for a visual check of product usage, and with their completed diary for review by study staff.

### Study visits and clinical procedures

This study was conducted at All Sum Research Center Ltd, Mississauga, Ontario, Canada. At screening (Visit 1), gingivitis and plaque accumulation were assessed, including an oral soft tissue (OST) and oral hard tissue (OHT) examination, dentition exclusions (missing teeth), and a gross gingival assessment. Before the baseline visit (Visit 2), which occurred within 10 to 28 days of screening, eligible participants abstained from oral hygiene for 12 h. During the visit, a full OST and OHT examination and assessments of gingival inflammation (MGI), gingival bleeding (BI), and supra-gingival plaque (TPI) were carried out. Prior to TPI assessment, dental plaque was stained using a disclosing dye solution (Chrom-O-Red). MGI was assessed at a total of four sites, and BI and TPI at a total of six sites, on the facial and lingual surfaces of each scorable tooth. Afterwards, in line with the current standard of care for people with gingivitis, a full dental prophylaxis was performed for each participant using periodontal instruments and a standard polishing dental compound prophylaxis paste followed by flossing by the clinician to remove sub- and supra-gingival calculus, stain, plaque and debris from the teeth [39]. A second clinician checked to ensure all plaque had been removed. Any remaining residual plaque was removed by the clinician, sufficient to bring the participant to zero plaque (i.e., TPI = 0), thereby reducing inter-participant variability in plaque levels at the start of the treatment period and enabling more reliable measurement of plaque removal efficacy at each of the following study visits. Eligible participants were randomized to study product and stratified based on gender and baseline NBS (low: < 45; high ≥ 45). Participants then underwent a supervised brushing, where they were instructed to brush for 1 timed minute at site with their assigned study product, after which they were instructed to continue using their product twice daily (morning and evening until their next visit). Participants returned to the study site at Day 3, Weeks 1, 2 and 6 (Visits 3, 4, 5, and 6 respectively) with overnight plaque at approximately the same time of day as the baseline visit and underwent a full OST examination followed by MGI, BI and TPI assessments. Adverse events (AEs) and incidents were recorded from informed consent and at the end of each study visit.

OST examinations were undertaken throughout the study by direct observation and palpation with retraction aids as appropriate and included examination of the labial mucosa (including lips), buccal mucosa, mucogingival folds, gingival mucosa, hard palate, soft palate, tonsillar area, pharyngeal area, tongue, sublingual area, submandibular area, and salivary glands. OHT examinations throughout the study were performed by direct observation and assessed the teeth including grossly carious lesions or signs of erosive wear, enamel irregularities, tooth fracture, gross decay, decalcification, faulty restorations, and implants. During the OST and OHT examinations, if any abnormalities were detected, the participant was advised to seek further medical/ dental advice from their dentist or general medical practitioner. A single suitably trained and calibrated dental examiner or clinically qualified designee was responsible for undertaking the OHT and OST examinations and assessing the gingivitis and plaque measures for the duration of the study for all participants.

### Study objectives

The primary efficacy endpoint was NBS at Week 6 of the experimental toothpaste compared with the negative control toothpaste. The secondary efficacy endpoint was NBS at Week 6 for the following comparisons: experimental toothpaste versus positive control toothpaste, positive control toothpaste versus negative control toothpaste. Exploratory efficacy evaluations included NBS at Day 3, and Weeks 1 and 2; mean BI; mean MGI and mean TPI (overall and interproximal) at Day 3, and Weeks 1, 2 and 6. All the exploratory efficacy endpoints were assessed for the following three comparisons: experimental toothpaste versus positive control toothpaste, experimental toothpaste versus negative control toothpaste and positive control toothpaste versus negative control toothpaste. Exploratory efficacy evaluations also included the comparison of MGI and mean BI in low (< 45 NBS) and high (≥ 45 NBS) BI subgroups over 6 weeks.

### Statistical analysis

The study plan enabled screening and randomization of a sufficient number of participants to ensure 36 evaluable participants per treatment group (108 total) completed the study. No formal study powering was conducted. The efficacy analysis was performed on a modified intent-to-treat (mITT) population, comprised of all randomized participants who received ≥ 1 dose of the study product and provided ≥ 1 post-baseline assessment of efficacy. Safety analysis was performed on the safety population, comprised of all randomized participants who received ≥ 1 dose of study product. All statistical tests of hypotheses were conducted at an unadjusted two-sided significance level of 0.05. This study was considered successful if a statistically significant difference between the adjusted mean NBS of the two study product groups (experimental toothpaste and negative control) at Week 6 was observed to be in favor of the experimental toothpaste. NBS at each time point was analyzed using an analysis of covariance (ANCOVA) model with study product group and gender as factors, and baseline NBS as covariate. Mean BI, MGI, overall TPI and interproximal TPI were analyzed using ANCOVA model with study product group, gender and NBS strata as factors. The covariate for mean BI was baseline BI score, for mean MGI was baseline mean MGI score, and for TPI was baseline plaque score. Adjusted means for all products and all pairwise product differences were provided along with 95% confidence intervals (CIs).

### Repeatability

To assess intra-examiner repeatability, at Visits 2, 3, 4, 5 and 6, repeatability data were generated for MGI and TPI assessments from replicate examinations on the same participant. At least two repeat assessments were performed for each index on each clinical assessment day. Repeatability examinations were separated by a minimum of 10 min and, where possible, separated by another participant. Due to the invasive nature of the BI assessment, it was not feasible to conduct an accurate repeatability assessment for this index.

MGI and TPI assessments on each tooth at each visit were cross-tabulated and a Fleiss-Cohen weighted Kappa coefficient (κ) was calculated, along with the 95% CI to assess the intra-examiner reliability. Repeatability was compared against pre-defined values as excellent if κ > 0.75; fair to good if 0.4 ≤ κ ≥ 0.75; and poor if κ < 0.4.

## Results

### Study population

**Fig. 1:**
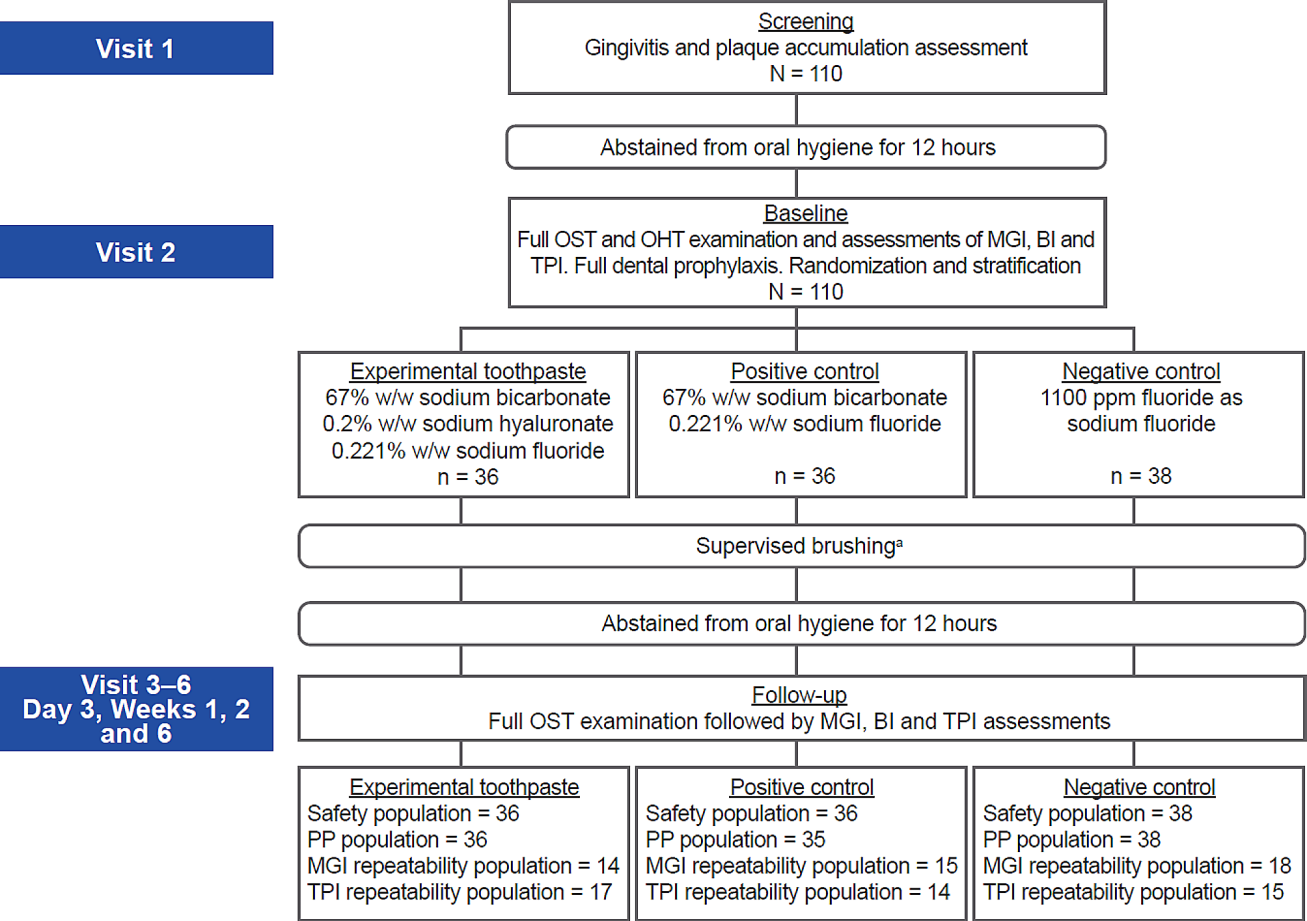
Study design. ^a^ Brushing at home > 12 h prior to each study visit was not supervised. BI, Bleeding Index; MGI, Modified Gingival Index; OHT, oral hard tissue; OST, oral soft tissue; PP, per protocol; TPI, Turesky Plaque Index; w/w, weight by weight

The first participant was enrolled on February 10, 2021, with the last participant completing on July 28, 2021. A total of 110 participants were screened for entry into the study, all of whom were enrolled and randomized to a treatment (36 participants in the experimental toothpaste group and positive control toothpaste group each, and 38 participants in the negative control toothpaste group). All the randomized participants completed the study (Fig. 1). Baseline characteristics were well balanced among treatment groups (Table 1).

**Table 1:** Demographic and baseline characteristics (safety population)

	Experimental toothpaste *n* = 36	Positive control toothpaste *n* = 36	Negative control toothpaste *n* = 38	Overall *N* = 110
**Male, n (%)**	14 (38.9)	14 (38.9)	16 (42.1)	44 (40.0)
**Mean age, years (range)**	37.9 (18–64)	41.5 (18–64)	40.3 (18–62)	39.9 (18–64)
**Race, n (%)**				
White African American/African heritage Asian Multiple	25 (69.4) 7 (19.4) 2 (5.6) 2 (5.6)	25 (69.5) 5 (13.9) 5 (13.9) 1 (2.8)	19 (50.0) 8 (21.1) 11 (28.9) 0	69 (62.7) 20 (18.2) 18 (16.3) 3 (2.7)
**Baseline mean BI score (SD)**	0.217 (0.0828)	0.224 (0.1166)	0.207 (0.1390)	0.216 (0.1148)
**Baseline mean MGI score (SD)**	2.115 (0.0721)	2.131 (0.0738)	2.099 (0.0879)	2.115 (0.0788)
**Baseline mean TPI score (SD)**	2.552 (0.2678)	2.501 (0.2861)	2.459 (0.2753)	2.503 (0.2766)
**Stratification, n (%)**				
Stratum 1: male, baseline NBS < 45 Stratum 2: male, baseline NBS ≥ 45 Stratum 3: female, baseline NBS < 45 Stratum 4: female, baseline NBS ≥ 45	13 (36.1) 1 (2.8) 17 (47.2) 5 (13.9)	12 (33.3) 2 (5.6) 18 (50.0) 4 (11.1)	14 (36.8) 2 (5.3) 18 (47.4) 4 (10.5)	39 (35.5) 5 (4.5) 53 (48.2) 13 (11.8)

BI, Bleeding Index; MGI, Modified Gingival Index; NBS, number of bleeding sites; SD, standard deviation; TPI, Turesky Plaque Index

### Efficacy

**Fig. 2:**
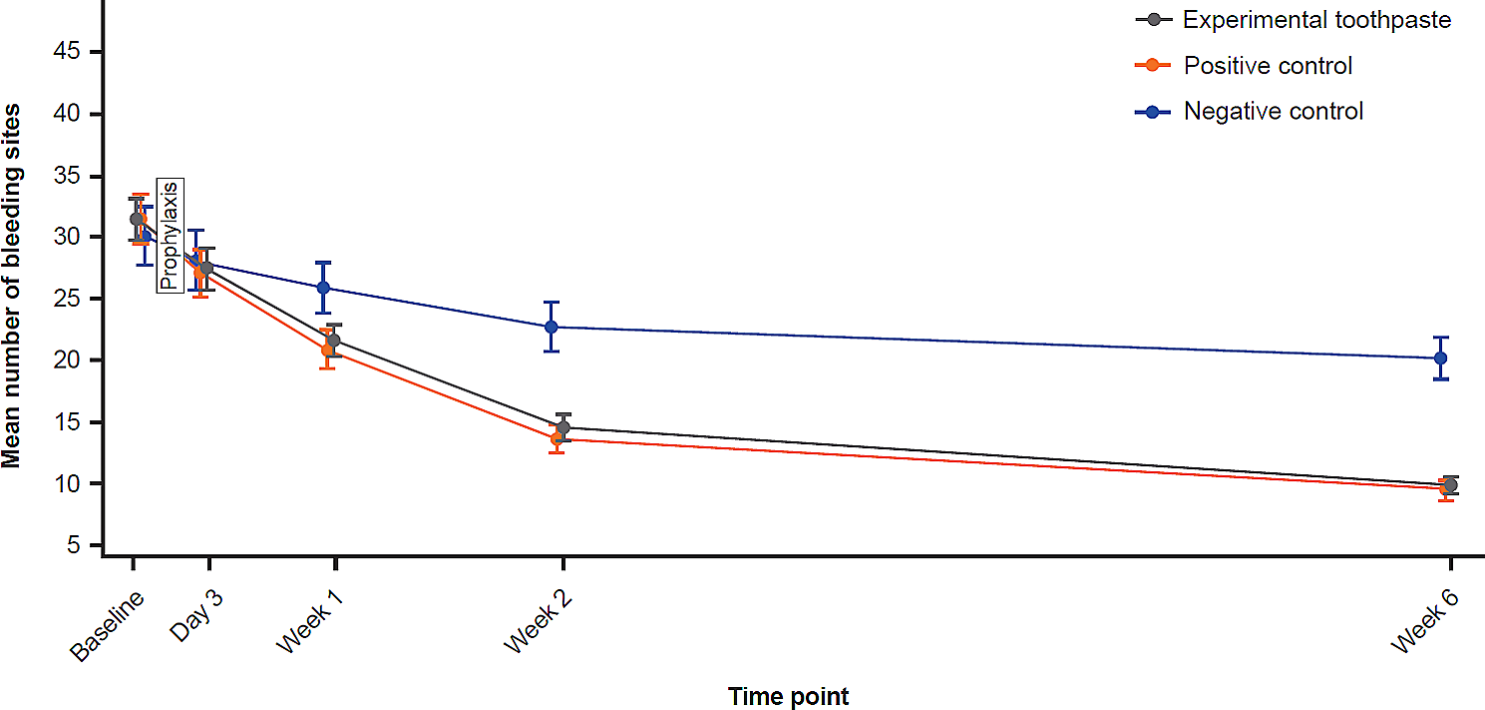
NBS over time for all study groups (mITT population). Error bars = standard error of the mean. NBS was calculated as the number of sites with BI score 1 or 2. mITT, modified intent-to treat; NBS, number of bleeding sites

The study met the primary endpoint with a significant reduction in the NBS for the experimental toothpaste group compared with the negative control toothpaste group after 6 weeks of treatment (adjusted means: 9.5 versus 20.5, respectively; adjusted mean difference: -11.0; 95% CI: -13.3, -8.8; percentage difference: -53.8%; *p* < 0.0001; Table 2; Fig. 2). No significant difference in NBS was observed in the experimental toothpaste group after 6 weeks compared with the positive control toothpaste group (adjusted means: 9.8 versus 9.4, respectively; adjusted mean difference: 0.4; 95% CI: -1.6, 2.4; percentage difference: 4.2%; *p* = 0.4488). A significantly lower NBS was observed in the positive control toothpaste group after 6 weeks compared with the negative control toothpaste group (adjusted means: 9.1 versus 20.6, respectively; adjusted mean difference: -11.5; 95% CI: -13.8, -9.1; percentage difference: -55.8%; *p* < 0.0001).

**Table 2:** NBS at Week 6 (mITT population): Primary and secondary endpoints

Comparison between groups^a^	Adjusted mean (SE)^b^	Adjusted mean difference (SE)^b^	95% CI^b^	*p*-value^b^	Percentage difference^c^	*p*-value^d^
**Experimental toothpaste (n = 36) versus** **negative control toothpaste (n = 38)**	9.5 (0.80) 20.5 (0.78)	-11.0 (1.12)	-13.3, -8.8	< 0.0001	-53.8	< 0.0001
**Experimental toothpaste (n = 36) versus** **positive control toothpaste (n = 36)**	9.8 (0.71) 9.4 (0.71)	0.4 (1.01)	-1.6, 2.4	0.6960	4.2	0.4488
**Positive control Toothpaste (n = 36)** **versus** **negative control toothpaste (n = 38)**	9.1 (0.85) 20.6 (0.82)	-11.5 (1.18)	-13.8, -9.1	< 0.0001	-55.8	< 0.0001

^a^ Difference is mean NBS for first product minus second product (experimental minus negative control; experimental minus positive control; and positive control minus negative control), such that a negative difference favors the first product (experimental or positive control). ^b^ Analysis was performed using ANCOVA model with study product group and gender as factors and baseline NBS as covariate. The estimates were based on separate ANCOVA model using only the data for corresponding comparison. ^c^ Percentage difference calculated as (adjusted mean difference/adjusted mean of comparison toothpaste)*100. ^d^*p*-value from Van Elteren test (non-parametric)

ANCOVA, analysis of covariance; CI, confidence interval; mITT, modified intent-to-treat; NBS, number of bleeding sites; SE, standard error

**Fig. 3:**
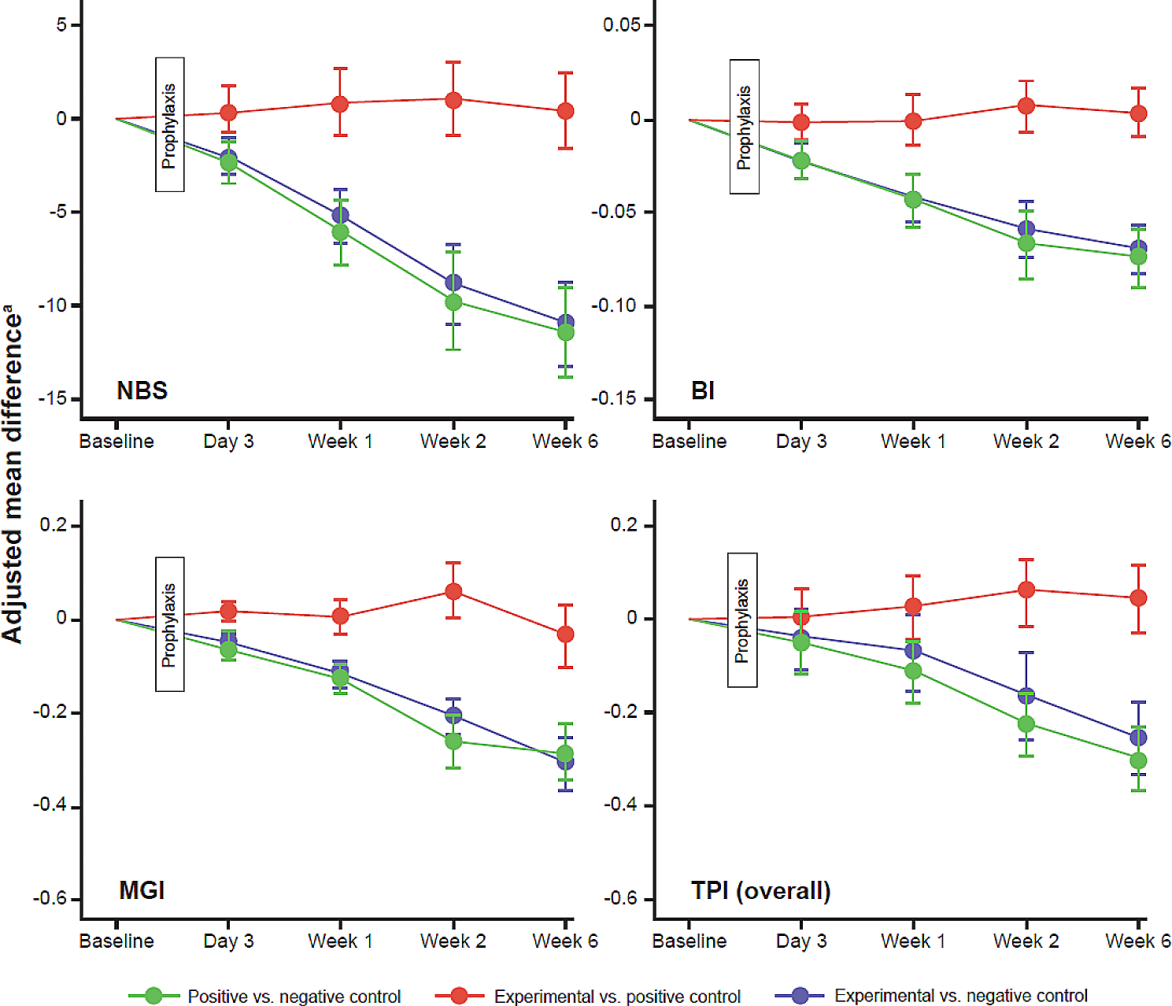
Summary of exploratory endpoints. Error bars = 95% CI. ^a^ Analysis was performed using ANCOVA model with the following factors: For NBS: study product group and gender as factors and baseline NBS as covariate; for BI: study product group, gender and NBS strata as factors, and baseline BI score as covariate; for MGI: study product group, gender and NBS strata as factors, and baseline MGI score as covariate; for TPI (overall): study product group, gender and NBS strata as factors and baseline overall TPI score as covariate. Difference is first product minus second product (experimental minus negative control; experimental minus positive control; and positive control minus negative control), such that a negative difference favors the first product (experimental or positive control). ANCOVA, analysis of covariance; BI, Bleeding Index; CI, confidence interval; MGI, Modified Gingival Index; NBS, number of bleeding sites; TPI, Turesky Plaque Index

Both sodium bicarbonate-containing toothpastes showed a reduction in adjusted mean NBS compared with the negative control at earlier time points too. For the experimental toothpaste versus negative control at Day 3, Week 1 and Week 2, the change in adjusted mean NBS was − 2.0 (95% CI: -3.0, -1.1), -5.2 (95% CI: -6.7, -3.8) and − 8.9 (95% CI: -11.1, -6.8), respectively. For the positive control versus negative control toothpastes at Day 3, Week 1 and Week 2, the change in adjusted mean NBS was − 2.4 (95% CI: -3.5, -1.3), -6.1 (95% CI: -7.8, -4.4) and − 9.8 (95% CI: -12.4, -7.2), respectively. The differences were significant except for the Day 3 time point, Fig. 3; Table 3. No difference in adjusted mean NBS was observed for the experimental toothpaste compared with the positive control toothpaste at any time point.

**Table 3:** Summary of statistical analysis for NBS, BI, MGI and TPI endpoints

Comparison between groups^a^	Parameter	Visit	Adjusted mean difference (SE)^b^	95% CI^b^	Percentage difference^c^	*p*-value^d^
**Experimental toothpaste (n = 36)** **versus** **negative control toothpaste (n = 38)**	NBS	Day 3 Week 1 Week 2 Week 6	-2.0 (0.48) -5.2 (0.75) -8.9 (1.07) -11.0 (1.12)	-3.0, -1.1 -6.7, -3.8 -11.1, -6.8 -13.3, -8.8	-7.1 -19.9 -38.8 -53.8	0.6284 0.0029 < 0.0001 < 0.0001
	BI	Day 3 Week 1 Week 2 Week 6	-0.023 (0.0049) -0.043 (0.0061) -0.059 (0.0075) -0.070 (0.0066)	-0.032, -0.013 -0.055, -0.030 -0.074, -0.044 -0.083, -0.057	-11.59 -24.35 -39.96 -54.57	0.9769 0.0105 < 0.0001 < 0.0001
	MGI	Day 3 Week 1 Week 2 Week 6	-0.048 (0.0095) -0.117 (0.0141) -0.205 (0.0201) -0.305 (0.0286)	-0.067, -0.030 -0.145, -0.089 -0.245, -0.165 -0.362, -0.248	-2.36 -5.77 -10.61 -16.58	0.0720 < 0.0001 < 0.0001 < 0.0001
	TPI (overall)	Day 3 Week 1 Week 2 Week 6	-0.042 (0.0321) -0.068 (0.0410) -0.161 (0.0466) -0.252 (0.0386)	-0.106, 0.022 -0.150, 0.014 -0.254, -0.068 -0.329, -0.175	-1.8 -3.0 -7.5 -11.9	0.7516 0.4290 0.0876 0.0008
	TPI (interproximal)	Day 3 Week 1 Week 2 Week 6	-0.048 (0.0348) -0.057 (0.0431) -0.131 (0.0463) -0.248 (0.0373)	-0.117, 0.022 -0.143, 0.029 -0.224, -0.039 -0.322, -0.174	-2.0 -2.5 -6.0 -11.4	0.7355 0.8315 0.2724 0.0006
**Experimental toothpaste (n = 36)** **versus** **positive control toothpaste (n = 36)**	NBS	Day 3 Week 1 Week 2 Week 6	0.4 (0.63) 0.8 (0.89) 1.0 (1.02) 0.4 (1.01)	-0.8, 1.7 -1.0, 2.6 -1.0, 3.0 -1.6, 2.4	1.5 3.8 7.2 4.2	0.5579 0.2009 0.5139 0.4488
	BI	Day 3 Week 1 Week 2 Week 6	-0.002 (0.0048) -0.001 (0.0067) 0.007 (0.0068) 0.003 (0.0063)	-0.012, 0.008 -0.014, 0.013 -0.007, 0.020 -0.009, 0.016	-1.10 -0.64 7.70 5.64	0.6531 0.8321 0.3952 0.3639
	MGI	Day 3 Week 1 Week 2 Week 6	0.021 (0.0109) 0.009 (0.0185) 0.066 (0.0295) -0.032 (0.0337)	-0.001, 0.042 -0.028, 0.046 0.007, 0.125 -0.100, 0.035	1.03 0.48 3.93 -2.03	0.7858 0.3416 0.1355 0.2630
	TPI (overall)	Day 3 Week 1 Week 2 Week 6	0.006 (0.0301) 0.026 (0.0343) 0.059 (0.0355) 0.045 (0.0361)	-0.055, 0.066 -0.043, 0.094 -0.012, 0.130 -0.027, 0.117	0.2 1.2 3.0 2.5	0.4707 0.0986 0.0299 0.1615
	TPI (interproximal)	Day 3 Week 1 Week 2 Week 6	0.008 (0.0337) 0.023 (0.0383) 0.063 (0.0352) 0.023 (0.0360)	-0.060, 0.075 -0.053, 0.100 -0.008, 0.133 -0.048, 0.095	0.3 1.0 3.1 1.2	0.3712 0.0729 0.0141 0.2069
**Positive control toothpaste (n = 36)** **versus** **negative control toothpaste (n = 38)**	NBS	Day 3 Week 1 Week 2 Week 6	-2.4 (0.57) -6.1 (0.86) -9.8 (1.29) -11.5 (1.18)	-3.5, -1.3 -7.8, -4.4 -12.4, -7.2 -13.8, -9.1	-8.3 -23.0 -42.6 -55.8	0.5544 0.0008 < 0.0001 < 0.0001
	BI	Day 3 Week 1 Week 2 Week 6	-0.022 (0.0050) -0.044 (0.0067) -0.067 (0.0093) -0.074 (0.0077)	-0.032, -0.012 -0.057, -0.030 -0.086, -0.049 -0.090, -0.059	-11.19 -24.57 -45.42 -57.27	0.8981 0.0104 < 0.0001 < 0.0001
	MGI	Day 3 Week 1 Week 2 Week 6	-0.065 (0.0091) -0.125 (0.0156) -0.262 (0.0265) -0.281 (0.0306)	-0.083, -0.046 -0.156, -0.093 -0.315, -0.209 -0.342, -0.220	-3.13 -6.12 -13.53 -15.18	0.0124 < 0.0001 < 0.0001 < 0.0001
	TPI (overall)	Day 3 Week 1 Week 2 Week 6	-0.048 (0.0333) -0.110 (0.0333) -0.220 (0.0340) -0.295 (0.0342)	-0.114, 0.019 -0.176, -0.044 -0.288, -0.152 -0.363, -0.227	-2.0 -4.8 -10.3 -14.0	0.6576 0.0390 < 0.0001 < 0.0001
	TPI (interproximal)	Day 3 Week 1 Week 2 Week 6	-0.053 (0.0367) -0.095 (0.0354) -0.192 (0.0359) -0.269 (0.0334)	-0.126, 0.021 -0.166, -0.025 -0.264, -0.120 -0.335, -0.202	-2.2 -4.2 -8.8 -12.5	0.7204 0.1379 < 0.0001 < 0.0001

^a^ Difference is mean NBS, BI, MGI and TPI for first product minus second product (experimental minus negative control; experimental minus positive control; and positive control minus negative control), such that a negative difference favors the first product (experimental or positive control). ^b^ Analysis was performed using ANCOVA model with the following factors: For NBS: study product group and gender as factors and baseline NBS as covariate; for BI: study product group, gender and NBS strata as factors, and baseline BI score as covariate; for MGI: study product group, gender and NBS strata as factors, and baseline MGI score as covariate; for TPI (overall): study product group, gender and NBS strata as factors and baseline overall TPI score as covariate; for TPI (interproximal): study product group, gender and NBS strata as factors and baseline interproximal TPI score as covariate

^c^ Percentage difference calculated as (adjusted mean difference/adjusted mean of comparison toothpaste)*100. ^d^*p*-value from Van Elteren test (non-parametric)

ANCOVA, analysis of covariance; BI, Bleeding Index; CI, confidence interval; MGI, Modified Gingival Index, NBS, number of bleeding sites; SE, standard error; TPI, Turesky Plaque Index

Both sodium bicarbonate-containing toothpastes showed a reduction in adjusted mean MGI compared with the negative control at each time point. For the experimental toothpaste versus negative control at Day 3, Week 1, Week 2 and Week 6, the change in adjusted mean MGI was − 0.048 (95% CI: -0.067, -0.030), -0.117 (95% CI: -0.145, -0.089), -0.205 (95% CI: -0.245, -0.165) and − 0.305 (95% CI: -0.362, -0.248), respectively. For the positive control versus negative control toothpastes at Day 3, Week 1 and Week 2, the change in adjusted mean MGI was − 0.065 (95% CI: -0.083, -0.046), -0.125 (95% CI: -0.156, -0.093), -0.262 (95% CI: -0.315, -0.209) and − 0.281 (95% CI: -0.342, -0.220), respectively. The differences were significant except for the Day 3 time point. No difference in MGI was observed for the experimental toothpaste, compared with the positive control toothpaste at any time point.

A summary of statistical analysis for NBS, BI, MGI and TPI endpoints is provided in Fig. 3; Table 3. BI, MGI, TPI (overall and interproximal) all favored both sodium bicarbonate-containing toothpastes (experimental and positive control) compared with a negative control at all measured time points, although, again, significant differences were not observed at the Day 3 time point. Both the experimental toothpaste and positive control toothpaste groups showed similar results for mean BI and MGI in both subgroups (low [< 45] and high [≥ 45] NBS), Table S1 and Table S2, respectively. Additionally, excellent reliability was observed for both MGI and TPI in the repeatability analysis, Table S3.

### Safety

There were no AEs, serious AEs, treatment-emergent AEs (TEAEs), or treatment-related TEAEs reported during the study and, therefore, no withdrawals due to TEAEs. No OST abnormalities were reported by examiners during the study.

## Discussion

This randomized control trial investigated the ability of an experimental toothpaste containing 67% w/w sodium bicarbonate, 0.2% w/w high molecular weight (HMW) sodium hyaluronate, and 0.221% w/w sodium fluoride to control mild-to-moderate gingivitis. Both the experimental toothpaste and the positive control (containing 67% w/w sodium bicarbonate and 0.221% w/w sodium fluoride) demonstrated improved gingival health compared with a regular fluoride toothpaste. However, no additional gingival health improvement was observed for the experimental toothpaste compared with the positive control. Therefore, no additional gingival health benefit can be attributed to the inclusion of sodium hyaluronate in this formulation.

Previous studies have demonstrated the efficacy of 0.2% w/w HMW sodium hyaluronate in a gel format for the treatment of gingivitis [28, 29]. This study showed that, while the experimental toothpaste could improve NBS after 6 weeks compared with the negative control, the addition of 0.2% HMW sodium hyaluronate in this formulation did not alter the efficacy, either positively or negatively, compared with the positive control toothpaste containing 67% w/w sodium bicarbonate and 0.221% w/w sodium fluoride. The findings of this investigation are consistent with prior studies that have shown the benefit of sodium bicarbonate-containing toothpastes for adults with gingival bleeding and inflammation after 6 weeks [10, 11, 18].

Hyaluronic acid was included in the experimental toothpaste as a primary film-forming polymer. The study was designed to test the hypothesis that sodium bicarbonate would remove plaque, while hyaluronic acid would create a film to prevent plaque build-up. This complementary mode of action would be expected to result in greater improvements in gingival health and plaque control, particularly with respect to plaque control at early time points. To observe these changes, this study carried out Modified Gingival Index (MGI), gingival bleeding (Bleeding Index [BI]), and supra-gingival plaque (Turesky Plaque Index [TPI]) assessments as early as 3 days, a time point not assessed in the previous gingivitis studies for sodium bicarbonate-containing toothpastes [10, 11, 18]. Statistically significant improvements in NBS, MGI and TPI were observed at 1 week for the positive control and experimental toothpastes compared with the negative control, when used twice daily and following a professional dental cleaning treatment. These data, therefore, confirm that use of toothpastes containing 67% sodium bicarbonate results in an early and sustained improvement of gingivitis compared with a regular fluoride-containing toothpaste, when used following a dental professional cleaning treatment. However, this study does not provide any evidence to suggest that hyaluronic acid enhances the plaque control and anti-gingivitis benefits of high-level sodium bicarbonate toothpastes with twice-daily use. In this study the percentage difference in NBS for the experimental toothpaste and the positive control versus the negative control after 2 weeks was − 38.8% and − 42.6% and after 6 weeks was − 53.8% and − 55.8%, respectively (both *p* < 0.0001). The observed differences in NBS are considered as clinically relevant improvements in gingival health [40].

All study toothpastes were well tolerated with no TEAEs, serious AEs, or medical device incidents reported during the study.

One limitation of this study was that. all participants underwent dental prophylaxis at the baseline visit; whilst in line with the recommended standard of care for people with gingivitis, this may be seen as a limitation as the results of the study should only be viewed in the context of patients who undergo or have access to the same standard of professional care [39].

Secondly, 0.2% w/w HMW sodium hyaluronate was not investigated alone, therefore, this study is limited in that it cannot comment on the anti-gingivitis efficacy of HMW sodium hyaluronate in the absence of an active compound. Furthermore, any potential additive benefits of HMW sodium hyaluronate would likely appear too small compared with the benefits of plaque control afforded by sodium bicarbonate toothpastes alone. However, the present results add to the existing body of evidence on sodium bicarbonate toothpastes, especially for use in plaque control at early time points.

## Conclusions

No additional anti-gingivitis efficacy (measured by NBS) was observed for the experimental toothpaste (containing sodium bicarbonate and sodium hyaluronate) compared with the positive control toothpaste (containing sodium bicarbonate but no sodium hyaluronate). Both the experimental and the positive control toothpastes demonstrated significant anti-gingivitis efficacy and reduced gingival bleeding compared with a regular fluoride toothpaste (negative control). All the study products were generally well tolerated.

### Electronic supplementary material

Below is the link to the electronic supplementary material.

Supplementary Material 1

## Data Availability

The datasets used and/or analyzed during the current study are available from the corresponding author on reasonable request. The full trial protocol is available at: https://clinicaltrials.gov/ct2/show/NCT04737538?term=208175&cond=Gingivitis&draw=2&rank=1.

## Abbreviations

AE: Adverse event
ANCOVA: Analysis of covariance
BI: Bleeding Index
CI: Confidence interval
HMW: High molecular weight
MGI: Modified Gingival Index
mITT: Modified intent-to-treat
NBS: Number of bleeding sites
OHT: Oral hard tissue
OST: Oral soft tissue
SD: Standard deviation
SE: Standard error
TPI: Turesky Plaque Index
w/w: Weight by weight
