# Supplementary material for: Efficacy of an experimental toothpaste containing sodium bicarbonate, sodium hyaluronate and sodium fluoride on gingivitis
Source: BMC Oral Health. 2024 Feb 9;24:209. doi: 10.1186/s12903-024-03981-9 (PMC10858478; doi:10.1186/s12903-024-03981-9)
Supplement: Supplementary file 1 — Supplementary Material 1 [file 12903_2024_3981_MOESM1_ESM.pdf]

Supplementary information for:

Efficacy of an experimental toothpaste containing sodium bicarbonate, sodium hyaluronate and sodium fluoride on gingivitis

Nisha Patel[1], Alyson Axe[1], Jimmy Qaqish[2], Martin R. Ling[1], Mako Araga[3], Charlie Parkinson[1], Chaju Ram Goyal[2]

[1]Haleon, Weybridge, Surrey, UK; [2]All Sum Research, Mississauga, Ontario, Canada; [3]Haleon, Warren, NJ, USA

Table S1 Summary of statistical analysis of mean MGI score within each subgroup of low and high NBS (mITT population)

Comparison between groups[a]	Subgroup	Visit	Adjusted mean difference (SE)[b]	95% CI[c]	Percentage difference[c]	p-value[d]
**Experimental toothpaste (n = 36) versus negative control toothpaste (n = 38)**	NBS low < 45	Day 3	-0.046 (0.0107)	-0.068, -0.025	-2.2	0.1129
		Week 1	-0.116 (0.0175)	-0.150, -0.081	-5.7	0.0002
		Week 2	-0.210 (0.0276)	-0.265, -0.155	-10.8	< 0.0001
		Week 6	-0.305 (0.0336)	-0.371, -0.238	-16.5	< 0.0001
	NBS high ≥ 45	Day 3	-0.049 (0.0242)	-0.097, -0.001	-2.4	0.3808
		Week 1	-0.113 (0.0395)	-0.192, -0.035	-5.6	0.0992
		Week 2	-0.165 (0.0623)	-0.289, -0.042	-8.6	0.0112
		Week 6	-0.295 (0.0760)	-0.446, -0.144	-16.0	0.0797
**Experimental toothpaste (n = 36) versus positive control toothpaste (n = 36)**	NBS low < 45	Day 3	0.022 (0.0109)	0.000, 0.043	1.1	0.7868
		Week 1	0.014 (0.0177)	-0.021, 0.049	0.7	0.4494
		Week 2	0.074 (0.0280)	0.018, 0.130	4.5	0.1945
		Week 6	-0.022 (0.0341)	-0.089, 0.046	-1.4	0.3249
	NBS high ≥ 45	Day 3	0.015 (0.0242)	-0.033, 0.063	0.7	0.9552
		Week 1	-0.006 (0.0395)	-0.084, 0.072	-0.3	0.5096
		Week 2	0.021 (0.0623)	-0.102, 0.145	1.2	0.4417
		Week 6	-0.053 (0.0759)	-0.204, 0.097	-3.3	0.5839
**Positive control toothpaste (n = 36) versus negative control toothpaste (n = 38)**	NBS low < 45	Day 3	-0.068 (0.0109)	-0.090, -0.047	-3.3	0.0272
		Week 1	-0.130 (0.0179)	-0.165, -0.094	-6.4	0.0003
		Week 2	-0.284 (0.0282)	-0.340, -0.228	-14.6	< 0.0001
		Week 6	-0.283 (0.0344)	-0.351, -0.215	-15.3	< 0.0001
	NBS high ≥ 45	Day 3	-0.064 (0.0241)	-0.112, -0.016	-3.1	0.2307
		Week 1	-0.107 (0.0394)	-0.186, -0.029	-5.3	0.0604
		Week 2	-0.187 (0.0622)	-0.310, -0.063	-9.7	0.0208
		Week 6	-0.242 (0.0758)	-0.392, -0.091	-13.1	0.0551

[a] Difference is mean MGI score for first product minus second product (experimental minus negative control; experimental minus positive control; and positive control minus negative control), such that a negative difference favors the first product (experimental or positive control). [b] Analysis was performed using ANCOVA model with study product group, gender and NBS strata as factors, baseline mean MGI score as a covariate and NBS strata into study product group as interaction. [c] Percentage difference calculated as (adjusted mean difference/adjusted mean of comparison toothpaste)*100. [d] p-value from Van Elteren test (non-parametric).

ANCOVA, analysis of covariance; CI, confidence Interval; mITT, modified intent-to-treat; MGI, Modified Gingival Index; NBS, number of bleeding sites; SE, standard error.

Table S2 Summary of statistical analysis of mean BI score within each subgroup of low and high NBS

Comparison between groups[a]	Subgroup	Visit	Adjusted mean difference (SE)[b]	95% CI[c]	Percentage difference[c]	p-value[d]
**Experimental toothpaste (n = 36) versus negative control toothpaste (n = 38)**	NBS low < 45	Day 3	-0.016 (0.0055)	-0.027, -0.005	-8.5	0.3179
		Week 1	-0.033 (0.0071)	-0.048, -0.019	-19.9	0.1005
		Week 2	-0.050 (0.0084)	-0.067, -0.034	-37.1	< 0.0001
		Week 6	-0.053 (0.0069)	-0.066, -0.039	-44.3	< 0.0001
	NBS high ≥ 45	Day 3	-0.051 (0.0128)	-0.077, -0.026	-21.8	0.0118
		Week 1	-0.087 (0.0166)	-0.120, -0.054	-39.2	0.0060
		Week 2	-0.096 (0.0197)	-0.135, -0.057	-45.7	0.0083
		Week 6	-0.154 (0.0162)	-0.186, -0.121	-82.8	0.0062
**Experimental toothpaste (n = 36) versus positive control toothpaste (n = 36)**	NBS low < 45	Day 3	-0.002 (0.0054)	-0.013, 0.008	-1.4	0.6390
		Week 1	0.002 (0.0071)	-0.012, 0.016	1.2	0.4651
		Week 2	-0.002 (0.0084)	-0.018, 0.015	-1.8	0.5787
		Week 6	0.006 (0.0069)	-0.008, 0.019	9.5	0.1635
	NBS high ≥ 45	Day 3	0.006 (0.0125)	-0.019, 0.030	3.1	0.0214
		Week 1	-0.009 (0.0163)	-0.042, 0.023	-6.5	0.2282
		Week 2	0.055 (0.0193)	0.017, 0.094	94.5	0.3792
		Week 6	-0.004 (0.0159)	-0.036, 0.027	-12.0	0.3242
**Positive control toothpaste (n = 36) versus negative control toothpaste (n = 38)**	NBS low < 45	Day 3	-0.014 (0.0054)	-0.025, -0.003	-7.3	0.7196
		Week 1	-0.035 (0.0070)	-0.049, -0.021	-20.8	0.0555
		Week 2	-0.049 (0.0083)	-0.065, -0.032	-36.0	< 0.0001
		Week 6	-0.058 (0.0069)	-0.072, -0.045	-49.2	< 0.0001
	NBS high ≥ 45	Day 3	-0.057 (0.0122)	-0.081, -0.032	-24.1	0.2307
		Week 1	-0.078 (0.0158)	-0.109, -0.047	-35.0	0.0384
		Week 2	-0.151 (0.0188)	-0.188, -0.114	-72.1	0.0075
		Week 6	-0.149 (0.0155)	-0.180, -0.118	-80.4	0.0054

[a] Difference is mean BI score for first product minus second product (experimental minus negative control; experimental minus positive control; and positive control minus negative control), such that a negative difference favors the first product (experimental or positive control). [b] Analysis was performed using ANCOVA model with study product group, gender and NBS strata as factors, baseline mean BI score as a covariate and NBS strata into study product group as interaction. [c] Percentage difference calculated as (adjusted mean difference/adjusted mean of comparison toothpaste)*100. [d] p-value from Van Elteren test (non-parametric).

ANCOVA, analysis of covariance; BI, Bleeding Index; CI, confidence Interval; NBS, number of bleeding sites; SE, standard error.

Table S3 Intra-examiner repeatability analysis of MGI and TPI

	Initial scores	Repeat scores							Kappa[c] (95% CI)
		Missing	0	1	2	3	4	5	
**MGI[a]**	**Missing**	128	0	0	0	0	0	-	0.950 (0.941, 0.958)
**n = 47**	**0**	0	0	0	0	0	0	-	
	**1**	0	0	878	43	0	0	-	
	**2**	0	0	23	4963	25	0	-	
	**3**	0	0	0	38	382	0	-	
	**4**	0	0	0	0	0	0	-	
**TPI[b]**	**Missing**	186	0	0	0	0	0	0	0.939 (0.930, 0.949)
**n = 46**	**0**	0	0	6	54	5	0	0	
	**1**	0	0	529	4	17	0	0	
	**2**	0	1	43	6484	33	0	0	
	**3**	0	0	0	53	2259	2	0	
	**4**	0	0	0	2	8	382	0	
	**5**	0	0	0	0	0	0	12	

[a] MGI - 0: Absence of inflammation; 1: Mild inflammation; slight change in color, little change in color, little change in texture of any portion of marginal or papillary gingival unit; 2: Mild inflammation; criteria as above but involving the entire marginal or papillary gingival unit; 3: Moderate inflammation; glazing, redness, edema and/or hypertrophy of marginal or papillary gingival unit; 4: Severe inflammation; marked redness, edema and/or hypertrophy of marginal or papillary gingival unit, spontaneous bleeding, congestion, or ulceration.

[b] TPI - 0: No plaque; 1: Separate flecks of plaque at the cervical margin; 2: Thin continuous band of plaque (up to 1 mm) at the cervical margin; 3: Band of plaque wider than 1 mm but covering < 1/3 of the tooth surface; 4: Plaque covering ≥ 1/3 but < 2/3 of the tooth surface; 5: Plaque covering ≥ 2/3 of the tooth surface.

[c] A weighted Kappa coefficient, along with the 95% CI is calculated to assess the intra-examiner reliability. Reliability will be deemed as Excellent if kappa > 0.75, Fair to good if kappa is between 0.4 and 0.75, and Poor if kappa < 0.4.

CI, confidence interval; MGI, Modified Gingival Index; TPI, Turesky Plaque Index.
